# Endometriosis of the Terminal Ileum: A Diagnostic Dilemma

**DOI:** 10.1155/2012/742035

**Published:** 2012-09-11

**Authors:** Kerem Karaman, Emel Ebru Pala, Umit Bayol, Ozlem Akman, Mustafa Olmez, Saime Unluoglu, Safak Ozturk

**Affiliations:** ^1^Department of Third General Surgery Clinic, Tepecik Teaching and Research Hospital, Gaziler Cad. Number: 468, Tepecik Eğitim ve Araştırma Hastanesi, Yenisehir, 35110 Izmir, Turkey; ^2^Department of Pathology, Tepecik Teaching and Research Hospital, 35110 Izmir, Turkey

## Abstract

Endometriosis is characterized by the presence of endometrial tissue consisting of glands and/or stroma located outside the uterus. Involvement of the terminal ileum is extremely rare. Preoperative distinction of ileal endometriosis from other diseases of the ileocecal region is difficult in terms of clinical presentation, symptomatology, radiological appearance, and surgical and pathological findings. We report a case initially diagnosed as Crohn's disease due to a longstanding diarrhea with subsequent intestinal obstruction, but finally diagnosed as ileal endometriosis by histopathological evaluation after resection of the involved segment.

## 1. Introduction

Endometriosis is a common condition with an unknown etiology that occurs particularly in women of reproductive age and is defined as ectopic foci of benign functional endometrial tissue. Some theories to explain how endometrial tissue moves to other sites include reflux of the endometrial tissue through the fallopian tubes during menstruation which results in implantation of these cells with subsequent growth on serosal surfaces of abdominal and pelvic organs; metaplastic transformation of pluripotential peritoneal mesothelium; migration of these cells through the lymphatic system or via hematogenous spread; development of the endometrial nodules from the metaplasia of mullerian remnants [1–4]. Up to 15% of menstruating women suffer from endometriosis. Ovaries, uterosacral ligaments, fallopian tubes, Douglas pouch, and pelvic peritoneum are the most involved sites, whereas the gastrointestinal tract, vagina, rectovaginal septum, round ligament and the inguinal hernia sac are less frequently affected. Endometriosis rarely affects extra-abdominal organs and tissues such as lung, urinary system, skin, and the central nervous system. Involvement of the terminal ileum is extremely rare comprising less than 7% of all gastrointestinal tract endometriosis [5]. We report a case initially diagnosed as Crohn's disease due to a longstanding diarrhea with subsequent intestinal obstruction, but finally diagnosed as ileal endometriosis by histopathological evaluation after resection of the involved segment.

## 2. Case Presentation

**Figure 1:**
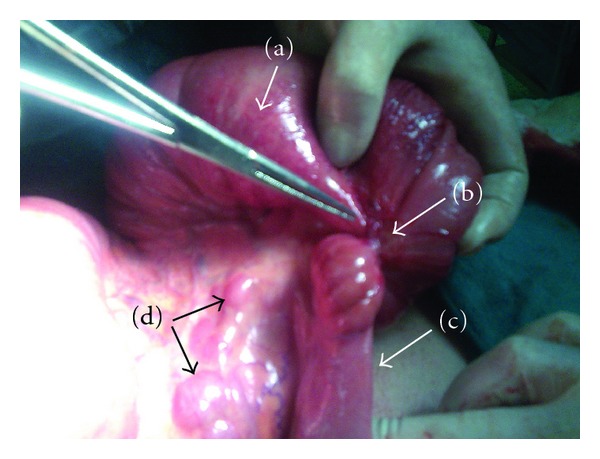
Image of the involved small bowel segment with endometriosis. (a) The proximal-dilated part of the terminal ileum. (b) The stricture caused by endometriosis. (c) The collapsed distal part of the terminal ileum. (d) Involved regional lymph nodes with endometriosis.

**Figure 2:**
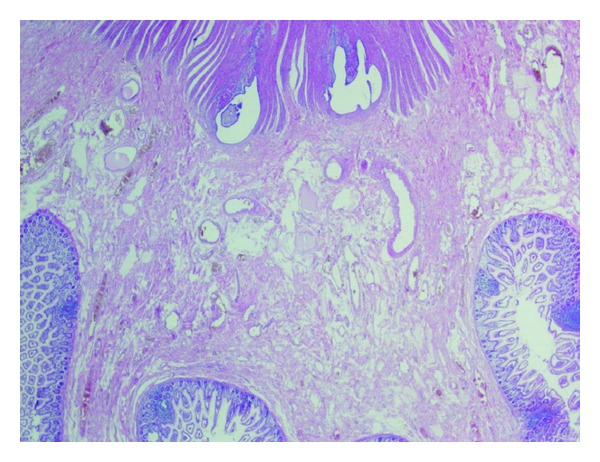
A nest of endometriotic glands and stroma lies in the muscularis propria of the ileum (HE, ×20).

**Figure 3:**
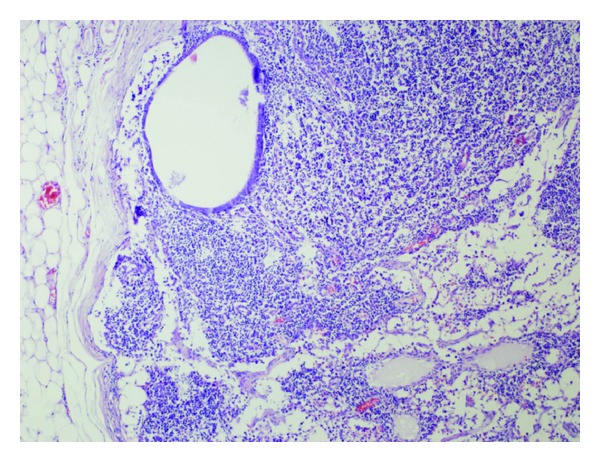
Endometriotic glands within lymph node (HE, ×100).

**Figure 4:**
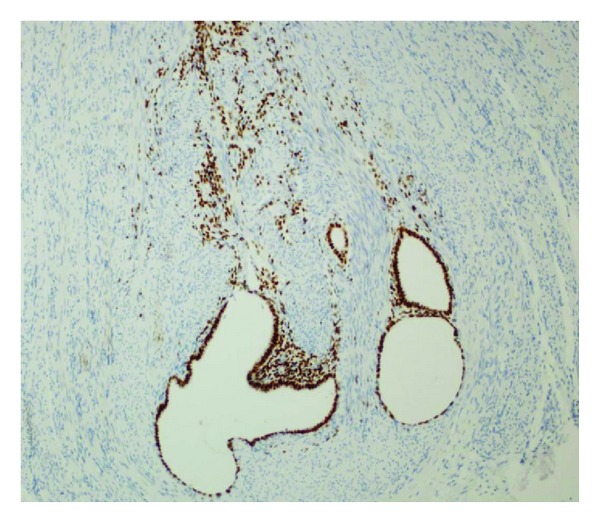
Both endometrial epithelial and stromal cells are highlighted by positive ER immunoreactivity (DAB, ×200).

**Figure 5:**
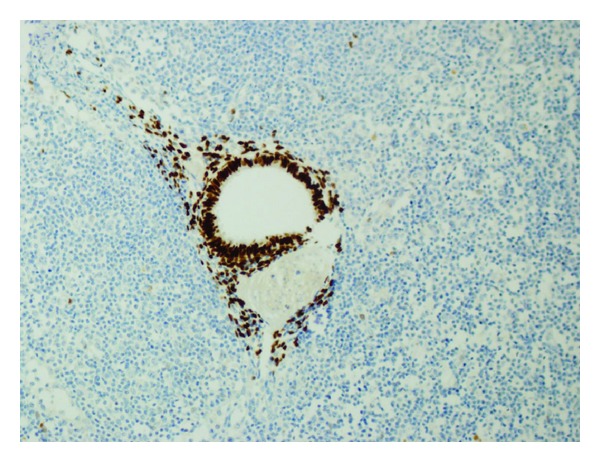
ER positivity of endometrial epithelial cells within lymph node (DAB, ×200).

A 27-year-old female patient was admitted to our gastroenterology clinic with complaints of abdominal pain and diarrhea of more than 3 months duration. The colonoscopic exam revealed inflammatory changes of the colonic mucosa suggesting inflammatory bowel disease. The computerized tomography showed a solid lesion measuring 2 cm in the left ovary in addition to dilatation of the small bowel segments. Hence, the patient was diagnosed as Crohn's disease and medication with salofalk and steroid treatment was introduced to treat the acute inflammatory attack. However, during her hospitalization period, she began to display progressive nausea and vomiting, suggesting that a small bowel obstruction had developed due to a stricture as a complication of the Crohn's disease. After consultation with the general surgery department on the patient's ileus, it was decided to perform an explorative laparotomy. The patient was explored and dilated small bowel segments, which ended 10 cm proximal to the cecum because a stricture was found (Figure 1). Multiple lymph nodes with a slightly thickened mesoperitoneum at the strictured ileal segment were palpated. Furthermore, an endometrial cyst 2 cm in diameter, originating from the left ovary, was also detected. The strictured ileal segment was resected and end-ileostomy was performed. Anastomosis was avoided because of the excessive dilatation of the proximal part of the ileum and suspicion of an inflammatory bowel which carries high risk for anastomotic leakage. The endometrial cyst was aspirated and a biopsy was taken from the cyst wall. Macroscopic examination of the resection specimen revealed dilated areas with normal bowel thickness and other areas that are firm and thickened. Microscopic examination revealed nests of endometriotic glands and stroma lying in the muscularis propria with regional lymph node involvement (Figures 2 and 3). The overlying mucosa was intact. Both endometrial epithelial and stromal cells were positive for estrogen receptor (ER) and progesterone receptor (PR) within ileal wall and lymph nodes (Figures 4 and 5). Stromal cells were also immunohistochemically CD10 positive. The histology of the ovarian cyst also showed endometriosis with endometriotic surface epithelial lining, cystically dilated endometrial glands, and numerous pigment laden histiocytes. The patient's postoperative period was uneventful, and after discharge she was referred to the gynecology outpatient department on medication for endometriosis.

## 3. Discussion

Endometriosis of the gastrointestinal tract is a common disorder that, when symptomatic, may be difficult to diagnose accurately. The rectosigmoid area (72%) is the most involved area. Other affected intestinal sites in decreasing order of frequency are the rectovaginal septum (13%), the small bowel (7%), the cecum (4%), and the appendix (3%) [6]. Involvement of the small bowel proximal to Meckel's diverticulum is extremely rare, but there are a few reported cases involving Meckel's diverticulum or jejunum [7, 8].

Intestinal endometriosis may present with a variety of symptoms that are commonly associated with other diseases. While intestinal symptoms may be exacerbated by menses, this association may not always be present as seen in our patient. When it does cause problems, the classic presentation is rectal bleeding at the time of menstruation. However, hemorrhage, intussusceptions, perforation, or small bowel obstruction may also occur [9]. The major cause of obstruction is stricture formation or adhesions, which can easily mimic Crohn's ileitis or a malignant mass. Other conditions that affect terminal ileum such as tuberculous enteritis, Yersinia enterocolitis, carcinoid tumors, small bowel lymphoma, and Behçet's disease should also be considered in differential diagnosis [10]. 

Small bowel obstruction due to ileal endometriosis is usually only being diagnosed at laparotomy and commonly causes diagnostic confusion with Crohn's disease. Both diseases are characterized grossly by patchy involvement of both the colon and the small intestine with intervening, uninvolved skip areas of the intestine. Enteric endometriosis is usually subserosal with less frequent involvement of the muscularis propria and submucosa. The mucosa is usually intact and uninvolved [11]. Although transmural involvement by Crohn's disease is the result of chronic injury, strictures and masses in endometriosis result largely from profound smooth muscle hypertrophy around endometriotic foci present in the muscularis propria. The “neurologic hypothesis” is a new concept in the pathogenesis of endometriosis which advocates that infiltration of the bowel wall is transmitted along the nerves, at a distance from the primary lesion [12]. Lesions originating from the serosa progressively invade the muscularis propria. The mucosa is rarely involved as it is poorly innervated. In addition, immunological, genetic, and familial aspects may also play a role in the pathogenesis of the disease. Other manifestations of Crohn's disease such as perianal abscesses or fistulas, and inflammatory pseudotumors involving the cecum, appendix, and terminal ileum have been described in intestinal endometriosis [13]. Microscopically endometriotic foci were composed of aggregates of small, often widely spaced endometrioid glands embedded within a variable amount of endometrial stroma [14]. Secretory endometrioed glands were not seen in any of the cases. Immunohistochemically, CD10 positivity—which indicates endometrial stroma—is a strong marker of endometriosis and can be used in the differential diagnosis [15].

Another characteristic aspect of the present case was the presence of endometrial stroma in regional lymph nodes (LN) of the resected small bowel segment. Endometriod lesions in regional LNs have been described in a number of reports with prevalence rates between 20% and 30% [16, 17]. The LN involvement was correlated with the number of LNs removed and size of the lesions [18]. There is no consensus regarding surgical removal of the involved lymph nodes. The basic surgical strategy for the treatment of endometriosis is to remove all visible endometriotic lesions. This strategy is based on the assumption that the removal of all lesions excludes the possibility of hormonal stimulation, reactivation, and renewed proliferation of endometriotic cells left in situ after surgery. On the other hand, the status of lymph nodes in endometriosis remains obscure because evidently lymph node dissection is usually not performed for benign disease. The addition of regional LN assessment increases the aggressiveness of interventions and thus potentially increases the morbidity [19].

Exact diagnosis of cases with no symptoms is difficult before surgery, and ultrasound, CT, and magnetic resonance imaging (MRI) may be of limited benefit. Endoscopic biopsies usually yield insufficient tissue for a definitive pathologic diagnosis as endometriosis involves the deep layers of the bowel wall. Diagnostic laparoscopy is the gold standard in detecting lesions. Biopsy of a suspicious area should be performed for precise diagnosis and for removal of lesions. 

The treatment of intestinal endometriosis consists of surgery and drug therapy. Hormone therapies with danazol or gonadotrophin-releasing hormone (GnRH) analogs are used in an attempt to eliminate residual endometriotic cells and reduce the risk of recurrence. However, although surgery with subsequent adjuvant medical therapy can effectively treat endometriosis in many women, the value of some adjuvant treatment strategies has been questioned and recurrence rates remain high [20].

In conclusion, preoperative distinction of ileal endometriosis from other diseases of the ileocecal region is difficult in terms of clinical presentation, symptomatology, radiological appearances, and surgical and pathological findings. However, ileal endometriosis should always be considered in the differential diagnosis in women of reproductive age.
